# Dipodal Silanes Greatly Stabilize Glass Surface Functionalization for DNA Microarray Synthesis and High-Throughput Biological Assays

**DOI:** 10.1021/acs.analchem.3c03399

**Published:** 2023-10-06

**Authors:** Arya Das, Santra Santhosh, Maya Giridhar, Jürgen Behr, Timm Michel, Erika Schaudy, Gisela Ibáñez-Redín, Jory Lietard, Mark M. Somoza

**Affiliations:** † Technical University of Munich, Germany, TUM School of Natural Sciences, Boltzmannstraße 10, 85748 Garching, Germany; ‡ Leibniz-Institute for Food Systems Biology at the Technical University of Munich, Lise-Meitner-Straße 30, 85354 Freising, Germany; § Technical University of Munich, Germany, TUM School of Life Sciences, Alte Akademie 8, 85354 Freising, Germany; ∥ Institute of Inorganic Chemistry, University of Vienna, Josef-Holaubek-Platz 2, 1090 Vienna, Austria; ⊥ Chair of Food Chemistry and Molecular Sensory Science, Technical University of Munich, Lise-Meitner-Straße 34, 85354 Freising, Germany

## Abstract

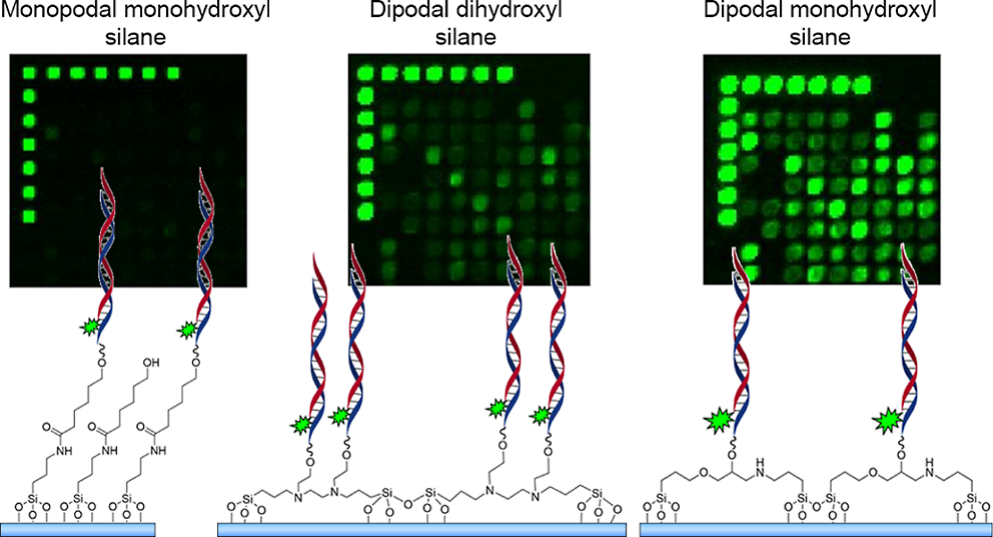

Glass is by far the most common substrate for biomolecular arrays, including high-throughput sequencing flow cells and microarrays. The native glass hydroxyl surface is modified by using silane chemistry to provide appropriate functional groups and reactivities for either in situ synthesis or surface immobilization of biologically or chemically synthesized biomolecules. These arrays, typically of oligonucleotides or peptides, are then subjected to long incubation times in warm aqueous buffers prior to fluorescence readout. Under these conditions, the siloxy bonds to the glass are susceptible to hydrolysis, resulting in significant loss of biomolecules and concomitant loss of signal from the assay. Here, we demonstrate that functionalization of glass surfaces with dipodal silanes results in greatly improved stability compared to equivalent functionalization with standard monopodal silanes. Using photolithographic in situ synthesis of DNA, we show that dipodal silanes are compatible with phosphoramidite chemistry and that hybridization performed on the resulting arrays provides greatly improved signal and signal-to-noise ratios compared with surfaces functionalized with monopodal silanes.

## 1 Introduction

Glass surfaces are almost universally used as supports for biomolecules in a wide range of high-throughput analyses, including sequencing flow cells,^1,2^ DNA arrays for gene expression studies,^3−5^ spatial transcriptomics analysis,^6^ DNA^7−10^ and RNA^11−13^ interactome analysis, aptamer-binding surveys,^14,15^ and epitope mapping using peptide arrays.^16,17^ Glass (generally borosilicate) is inexpensive and widely available and has physical and chemical properties that are often experimentally important including optical transparency, dimensional stability, inertness, and low autofluorescence. The chemical versatility of the silane chemistries adds synergistically to the desirable properties of glass, providing an accessible approach to efficiently derivatize the native hydroxyl groups with a wide variety of functional groups that can be used to create well-defined surface properties, including providing reactive groups suitable for immobilizing biological macromolecules and reactive groups suitable for in situ chemical peptide or nucleic acid synthesis.^18,19^ In parallel to immobilization chemistries, silanes can also be used to tune the hydrophobicity of the surface, e.g., for promoting tissue-to-surface coupling in spatial transcriptomics,^6^ or to provide antifouling properties that can reduce nonspecific adhesion of biomolecules to the surface.^19−21^

The primary disadvantage of silane-functionalized glass is the hydrolytic instability of the siloxy bond with the native glass surface’s hydroxyl groups.^22−24^ Around room temperature, at near-neutral pH, and for a few hours, the loss of siloxy bonds is low to moderate but increases rapidly with the higher temperatures and incubation times common in biological assays, often 1 to 2 days for high-throughput genomics experiments such as gene expression analysis and sequencing-by-synthesis. Loss of the surface-bound biomolecules increases with time and temperature, significantly reducing both signal and signal-to-noise ratios in experimental results while also preventing array reuse. To improve array stability, several carbon-based substrates have been successfully developed and validated for both low-density spotted DNA arrays and high-density DNA arrays synthesized in situ by photolithography. These carbon-based substrates include diamond films,^25−27^ glassy and amorphous carbon,^27−29^ carbon-on-metal,^30^ and polymers^31−33^ and are functionalized with a variety of chemistries that result in biomolecule immobilization via stable carbon–carbon bonds.^29,34−39^ Although these substrates provide superior stability, they are less accessible and less versatile than silanized glass substrates.

Here, we show that the substitution of conventional monopodal silanes with dipodal silanes—silanes with two silicon atoms each derivatized as trimethoxysilyl functions—results in a large increase in the stability of functional glass substrates. We demonstrate the utility of these substrates in the context of the in situ photolithographic synthesis of complex DNA arrays. The enhanced stability of the DNA on these arrays results in significantly improved signal-to-noise ratios in high-throughput genomics experiments. Specifically, we compared *N*-(3-triethoxysilylpropyl)-4-hydroxybutyramide (“monopodal monohydroxyl silane”), which has a 25 year history of use in functionalizing glass for in situ microarray synthesis,^40−42^ with two analogous but dipodal silanes, *N*,*N*′-bis(2-hydroxyethyl)-*N*,*N*′-bis(trimethoxysilylpropyl)-ethylenediamine and 1,11-bis(trimethoxysilyl)-4-oxa-8-azaundecan-6-ol, the former bearing two hydroxyl moieties (“dipodal dihydroxyl silane”) and the latter a single hydroxyl group (“dipodal monohydroxyl silane”) (Table 1) that can serve as initiation sites for DNA synthesis. Dipodal silanes are less well-known chemicals in the surface modification toolbox, having been used primarily to promote adhesion between organic and inorganic materials in environmental contexts that are challenging for monopodal silanes, such as marine coatings and dental fillings, where they provide high-density cross-links that improve hydrolytic stability, enhanced wet adhesion, chemical resistance, and mechanical strength.^43−46^ The reasons for the enhanced hydrolytic stability of dipodal silanes are not fully understood, but have been hypothesized to include more opportunities to form siloxane bonds to the surface and the formation of highly entangled polymeric silane films that restrict the accessibility of water to the surface.^43,46^ Nevertheless, already in 1998, McGall patented^47^ for Affymetrix the utility of dipodal silanes in the context of their improved hydrolytic stability under hybridization conditions on DNA arrays synthesized via photolithography. Although the data in the patent were never published in the scientific literature, we can confirm and extend the results, demonstrating an approximately 3- to 12-fold signal improvement and a 2- to 4-fold signal-to-noise improvement upon hybridization with fluorescently labeled complementary DNA to microarrays synthesized on glass slides functionalized with dipodal versus monopodal silanes, depending on the identity of the silane, the length of the hybridization, and the assessment metric. These results demonstrate that the sensitivity of high-throughput analyses on microarrays can be greatly improved by the use of dipodal silanes.

**Table 1:**
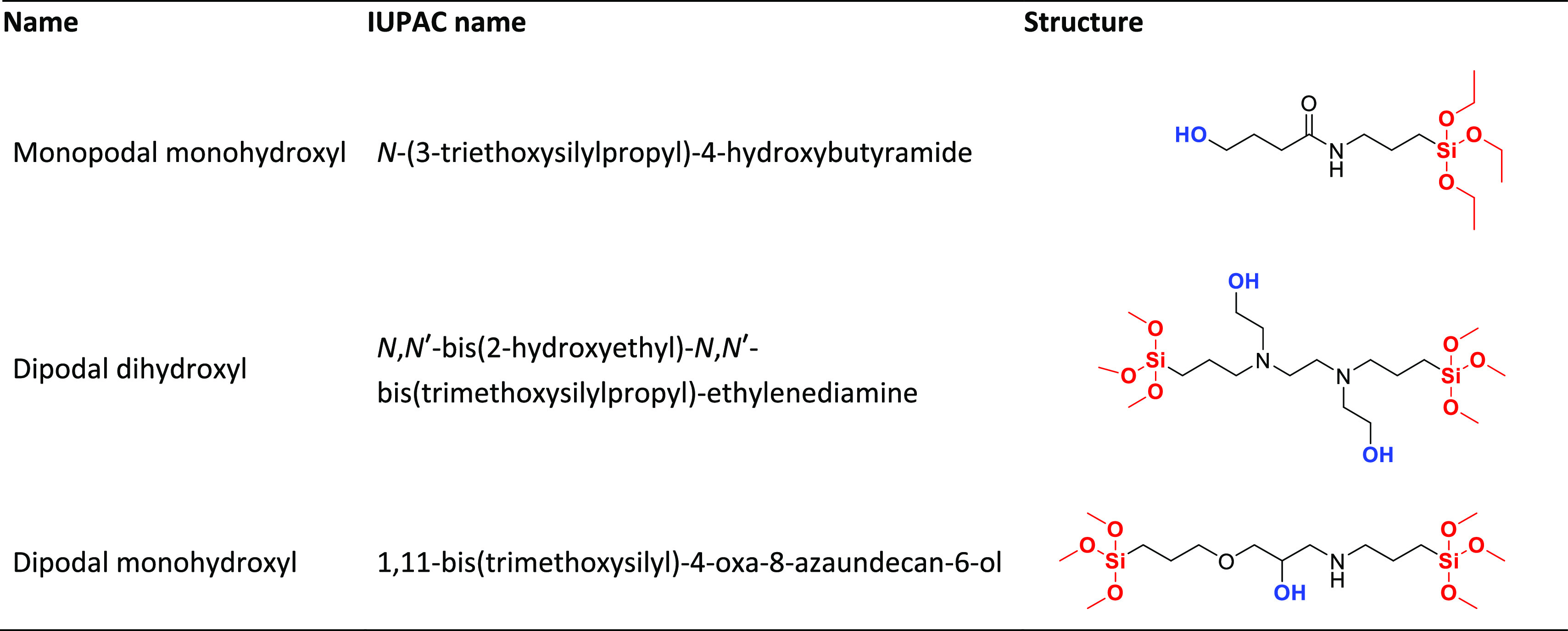
Silanes Were Used to Functionalize the Glass Substrates

## 2 Methods

### 2.1 Silanization of Glass Slides

All experiments used Schott Nexterion D 263, cleanroom-cleaned (Schott 1095568) borosilicate microscope slides. The glass microscope slides were functionalized with a solution consisting of a 95:5 (*v*/*v*) solution of ethanol/deionized water, plus 0.2% acetic acid and 32.5 mmol silane. Arranged in a stainless-steel rack (Sigma-Aldrich Z710989), the slides were immersed in the silane solution for a period of 4 h at room temperature under gentle agitation. The three different silanes that were used are listed in Table 1. The glass slides were then washed twice for 20 min in solutions containing 95:5 (*v*/*v*) EtOH/H_2_O and 0.2% acetic acid, drained of the wash solution, and gently blown with argon to remove residual droplets. The slides were then dried in a vacuum oven at 120 °C for 2 h and left to cool down overnight under vacuum. Functionalized slides were stored in a desiccator until further use.

### 2.2 Photolithographic Microarray Synthesis

A maskless array synthesizer, which consists of an optical system synchronized with a chemical delivery system, was used to generate DNA microarrays by means of in situ chemical photolithography. The synthesis was performed as previously published.^48−51^ Briefly, a 365 nm UV LED^52^ was used as the light source, and a calibrated intensity meter (Ushio UIT 201) was used to adjust the UV light intensity that is directed to the synthesis area to 100 mW/cm^2^. A chemical fluidics system (Expedite 8909 nucleic acid synthesizer), which transports solvents and chemicals to the photochemical flow cell, was synchronized by a computer with a sequence of virtual masks used to pattern the UV light. In the presence of a weak amine base (1% imidazole in dimethyl sulfoxide), the BzNPPOC (benzoyl-2-(2-nitrophenyl)-propoxycarbonyl)^49^ photolabile protecting group at the 5′ termini of the nascent oligonucleotides was selectively removed at positions exposed to 365 nm light. This left the 5′-hydroxyl groups available for coupling with the next 5′-BzNPPOC DNA phosphoramidite (Orgentis). The rest of the synthesis procedure was similar to that of conventional automated solid-phase oligonucleotide synthesis.^53^ After synthesis, the microarrays were immersed in a 1:1 (*v*/*v*) ethylenediamine/ethanol solution for 2 h at room temperature to remove the phosphodiester and nucleobase protecting groups. The slides were cleaned twice with deionized water, dried with argon, and stored in a desiccator until use.

### 2.3 Hybridization, Data Extraction, and Analysis

After synthesis, the microarrays were hybridized in SecureSeal hybridization chambers (Grace Bio-Labs SA200) at 42 °C with rotation using 350 μL of hybridization solution (175 μL of 2× MES buffer, 7.76 μL of acetylated bovine serum albumin (BSA) (20 mg/mL), 3.5 μL of 1 μM Cy3-labeled complementary DNA oligonucleotide, and 163.7 μL of deionized water), resulting in a final concentration of 10 nM complementary oligonucleotide. Depending on the experiment, the microarrays were hybridized for either 2 or 24 h. After hybridization, the chamber was removed and the microarrays were washed in 30 mL of nonstringent wash buffer (6× SSPE, 0.01% Tween 20) for 2 min, stringent wash buffer (100 mM MES, 0.1 M Na^+^, 0.01% Tween20) for 1 min, and final wash buffer (0.1× SSC) for a few seconds, all in 50 mL Falcon tubes. Slides were immediately dried in a microarray centrifuge and then scanned in a microarray scanner (Genepix 4400A, Molecular Devices) at an excitation wavelength of 532 nm, 100% laser power, a PMT voltage of 350 V, and a resolution of 5 μm. Data were extracted from the scanned images using NimbleScan 2.1 (Roche-NimbleGen) in the form of probe files containing the intensity of each array probe. The intensity values were then averaged across on-array replicates and replicate arrays and plotted as box-and-whisker plots using Origin 2018b. The data on hydrolytic stability during hybridization are based on six replicate arrays per silane treatment and 350 replicate probes per sequence length on each array. The data on linker-length dependence are based on two replicate arrays per silane treatment and 350 replicate probes per linker length on each array. The analysis of the gene expression array data is described below.

### 2.4 Spectrophotometric Surface Density Measurements

Surface DNA density was estimated by adapting the protocol described by Phillips et al.^27^ For this, a DNA 25mer (5′-GTCATCATCATGAACCACCCTGGTC-3′; “QC 25mer”) on a 5 nt dT linker was synthesized over the entire synthesis area (1.1 × 1.4 cm), and the arrays were hybridized to a Cy3-labeled complementary oligonucleotide by incubation with rotation for 2 h at 42 °C. The hybridization solution consisted of 150 μL of 2× MES buffer, 13.3 μL of acetylated BSA (10 mg/mL), 133.5 μL of 100 nM Cy3-labeled complementary oligonucleotide, and 3 μL of nuclease-free water (concentration of the labeled probe: 44.5 nM). Labeled DNA bound nonspecifically to the surface was removed by washing the arrays for 2 min in a nonstringent wash buffer, 1 min in stringent wash buffer, and 10 s in the final wash buffer. After drying, the synthesis area was covered with a hybridization chamber (Grace Bio-Labs SecureSeal SA200), and the arrays were incubated with 300 μL of wash-off buffer (40 mM KCl, 132 mM KOH) with rotation for 30 min at room temperature. The solution was collected and diluted to 600 μL with a wash-off buffer. The calibration solutions containing the Cy3-labeled complementary oligonucleotide (1 × 10^–11^ to 5 × 10^–8^ M) were also prepared in the wash-off buffer. The fluorescence signals of all samples were measured in triplicate by using a multimode microplate reader (TECAN Spark). The amount of washed-off DNA for each sample was calculated according to the calibration curve and served as an estimate for the density of hybridized on-array DNA.

### 2.5 Gene Expression Analysis

Ten μg of human reference total RNA (Agilent) was simultaneously reverse transcribed and labeled as described by Ouellet et al.^54^ Briefly, 18.5 μL of a solution containing 10 μg of total RNA, 7 μg of 5′-Cy3-labeled random nonamers (Biomers), and 1.5 μL of nuclease-free water was heated to 70 °C for 5 min for heat denaturation. After 5 min, the solution was immediately cooled on ice. 13.5 μL of reverse-transcription buffer was prepared by mixing 6 μL of 5× first-strand buffer (Invitrogen), 1.5 μL of 0.1 M DTT (Invitrogen), 1 μL of 10 mM dNTP (Thermo Scientific), 4 μL of 200 U/μL superscript III (invitrogen), and 1 μL of 40 U/μL RNaseOUT (Invitrogen). To this, the denatured solution was added and incubated at 25 °C for 5 min, followed by a 3 h incubation at 42 °C. For the hydrolysis reaction, 200 mM NaOH and 200 mM EDTA were taken in a 1:1 ratio, and 32 μL of the solution was added to the previous mixture. This was then incubated at 65 °C for 10 min 64 μL of 1 M HEPES at pH 7 (Carl Roth) was added to the mixture to neutralize the reaction solution. Purification was performed using a QiaQuick PCR purification kit (Qiagen) following the manufacturer’s protocol. The amount of cDNA was measured using a NanoDrop One (ThermoFisher Scientific). The yield of labeled cDNA was estimated to be 40%.

In the gene expression analysis experiments, 5 μg of Cy3-labeled cDNA was used per array. The hybridization solution was prepared by mixing 150 μL of 2× MES buffer, 3.33 μL of 10 mg/mL herring-sperm DNA (Promega), 16.67 μL of 10 mg/mL acetylated BSA (Promega), and equal volumes (11.11 μL each) of 100 nM solutions of three quality control synthetic Cy3-labeled oligonucleotides: “QC 25mer”, “ECO1BioA1”, and “ECO1BioD2”. Five μg of labeled cDNA in nuclease-free water was added, and the solution was brought to 300 μL with nuclease-free water. After self-adhesive hybridization chambers (Grace Bio-Labs SecureSeal SA200) were applied over the arrays, the hybridization buffer was pipetted into the chambers, which were sealed using stickers and incubated at 42 °C for 20–24 h with rotation. The slides, with hybridization chambers still attached, were immersed in 50 mL nonstringent wash buffer at 42 °C in a Petri dish. The hybridization chambers were removed while still immersed in the buffer to disperse the hybridization solution. The slides were immediately transferred to 50 mL Falcon tubes for sequential washing in nonstringent wash buffer for 2 min, stringent wash buffer for 1 min, and final wash buffer for a few seconds. The slides were dried using a microarray centrifuge and then scanned at 2.5 μm resolution with a PMT voltage of 350 V. Intensity data from the scanned images were extracted and analyzed using NimbleScan 2.1 (Roche-NimbleGen), employing robust multichip analysis (RMA^55^) for interarray normalization purposes. The gene expression data analysis is based on two arrays (control A and control B) per scatterplot. Each gene expression array contains three unique 60mer probes for each of 45,033 human genes.

## 3 Results and Discussion

### 3.1 Hydrolytic Stability during Hybridization

**Figure 1:**
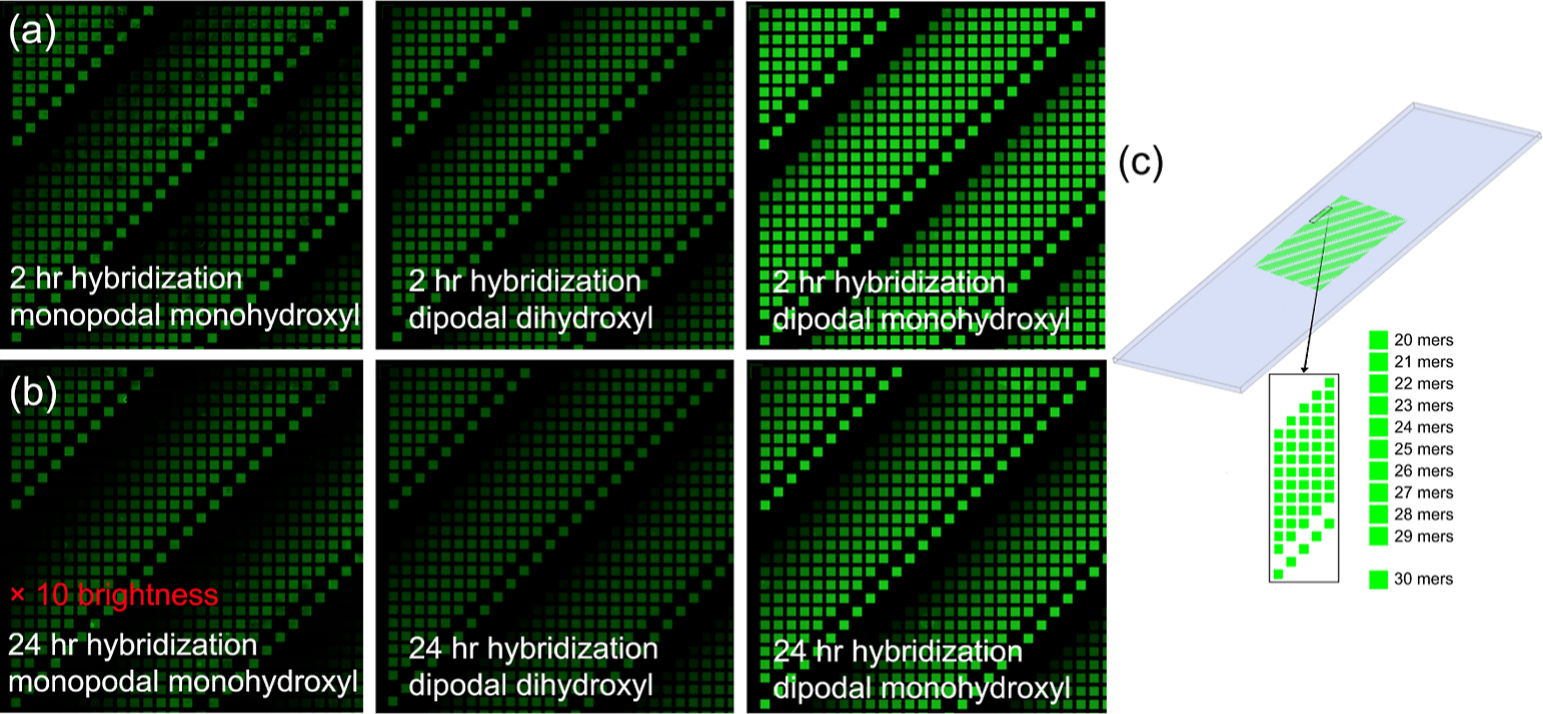
Fluorescent scan images of microarrays synthesized on glass substrates functionalized with the monopodal monohydroxyl, dipodal dihydroxyl, or dipodal monohydroxyl silanes and hybridized at 42 °C for 2 (a) or 24 h (b). The intensity of the image of the 24 h hybridization on the monopodal silane substrate has been increased by *a* factor of 10 to make the pattern visible. The layout and identity of the 20 through 30mers are illustrated in (c). All six array images were acquired with identical scanner settings (100% laser intensity, 350 V PMT).

To assess the hydrolytic stability of the DNA on microarrays synthesized on dipodal-versus monopodal-functionalized silanes, we synthesized DNA microarrays on glass surfaces functionalized with each silane. Using in situ photolithographic DNA synthesis, we generated simple microarrays with 11 sequences: a 30mer (5′-GTCATCATCATGAACCACCCTGGTCTTTTT-3′) and 10 stepwise truncations of this sequence from the 5′ terminus. After synthesis, the microarrays were hybridized for either 2 or 24 h with a Cy3-labeled oligonucleotide complementary to the full-length sequence on the microarray. Green-colored fluorescent images of representative arrays functionalized with the three silanes are shown in Figure 1. In addition to the silane treatment, we initially also experimented with the use of piranha solutions (5:1 ratio of sulfuric acid/hydrogen peroxide) to increase the surface hydroxyl density of the glass slides. Although piranha solutions have frequently been used prior to silanization, including in the context of in situ synthesis of DNA arrays,^42,56,57^ we observed only a few-percent improvement in fluorescence signal due to the piranha treatment in preliminary experiments and decided to omit this step in all experiments due to its safety risks. Therefore, all experiments presented here are based on slides that were silanized without any prior chemical oxidation or cleaning steps.

After the 2 h hybridizations, the substrates functionalized with monopodal monohydroxyl and dipodal dihydroxyl silanes exhibit similar fluorescence intensity patterns, but the fluorescence on the dipodal monohydroxyl silane was significantly higher. Additional arrays were hybridized in exactly the same manner but for 24 h. All three substrate types exhibited a decrease in fluorescence intensity relative to the 2 h hybridization, which we attribute to a loss of DNA probes due to hydrolysis of the siloxy bonds and subsequent detachment of DNA from the glass surface. In the case of the monopodal monohydroxyl silane, the loss is very high and requires digitally increasing the brightness of the image by *a* factor of 10 relative to the other images to render the spots visible (Figure 1b).

**Figure 2:**
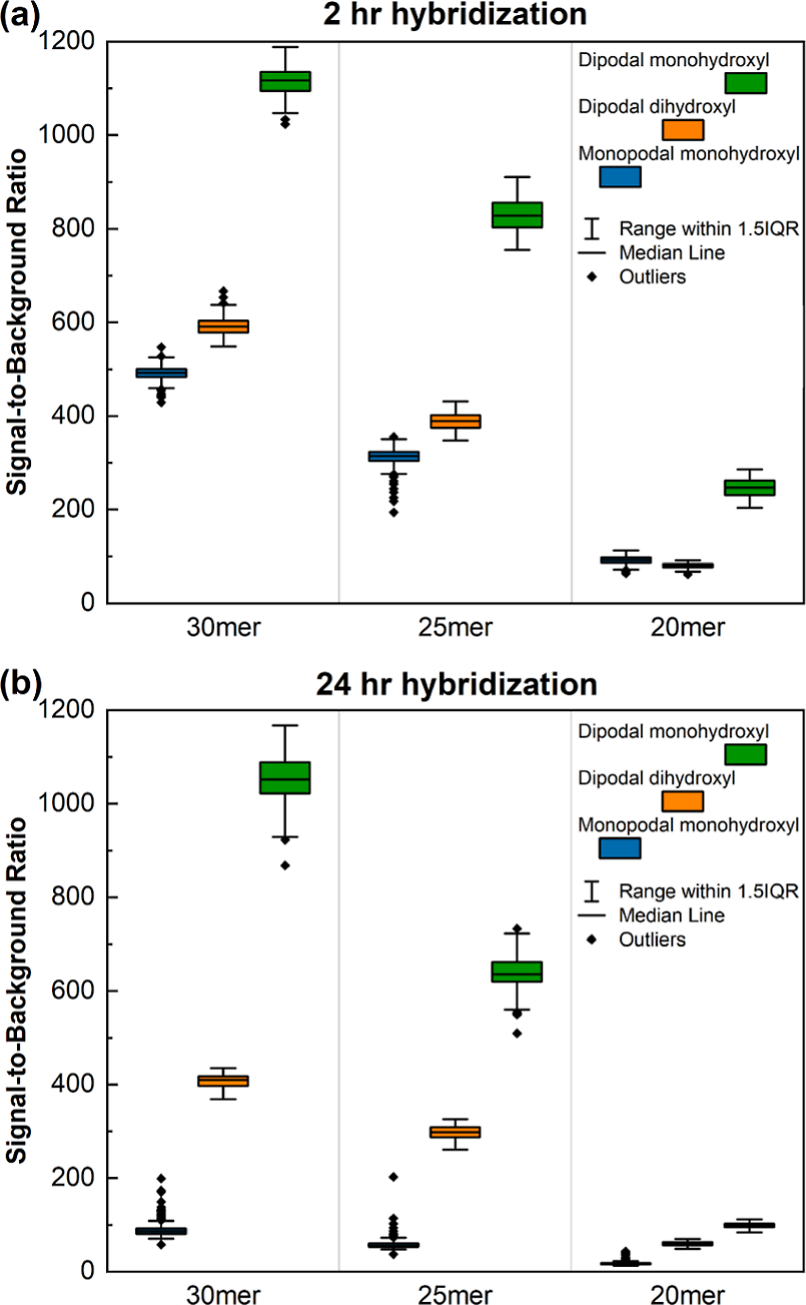
Signal-to-background ratio of hybridization-based fluorescence intensities of 30mer, 25mer, and 20mer sequences. Intensities measured after 2 (A) and 24 h (B) of hybridization of the monopodal monohydroxyl (blue), dipodal dihydroxyl (orange), and dipodal monohydroxyl (green) silane functionalized microarrays. Each boxplot is based on 350 replicates per sequence per array and six replicate arrays per silane.

To quantify the loss of DNA from the surface, we extracted the fluorescence intensity from all 350 on-array replicate features for each oligonucleotide length from each of the six replicate arrays for each silane surface functionalization. For each silane, the equivalent probes on each of the six arrays, a total of 2100 replicates, were averaged across the arrays to generate 350 data points per probe length. These data for both 2 and 24 h hybridizations and for the 30mers, 25mers, and 20mers are plotted in Figure 2. The data, expressed as the signal divided by the background (unhybridizable array areas), show that functionalization with both the dihydroxyl and monohydroxyl dipodal silane results in significantly higher signal intensities, relative to the monopodal silane, for all probe lengths except for the shortest (20 nt) in the case of the dihydroxyl dipodal silane. Expressing the hybridization-intensity data as signal-to-background ratios accounts for possible differences in nonspecific binding to the surface, but no significant differences in background intensity were observed, with typical values of 30 to 50 units of arbitrary intensity for the hybridization experiments performed in this work.

The higher signal intensities of the longer oligonucleotides are due to their higher duplex stability. We hypothesized that longer probes would be more likely to be lost from the surface with increased hybridization times as they would experience greater hydrodynamic forces that could potentially pull them off the surface if the silane bond is weak. This does not appear to be the case, as the presumed weaker surface bond of the monopodal monohydroxyl silane does not result in a differential loss of the 30mers vs 20mers between short and long hybridizations (Table 2). While the dipodal dihydroxyl silane surface also shows little length-dependent loss of DNA, the dipodal monohydroxyl silane does, but in the opposite direction, with a proportionally stronger hybridization signal for the 30mers vs 20mers after the longer hybridization. A possible explanation for this effect could be a higher initial surface density of available hydroxyl groups, resulting in a surface density of DNA probes that is too high to fully hybridize due to molecular crowding but which becomes less crowded due to DNA losses during the 24 h hybridization. In this case, the reduced crowding partially compensates for the DNA loss. The issue of the surface density of DNA synthesized on these substrates is explored below. This would imply that the 20mers are a better indicator of DNA loss from the surface, as they hybridize too poorly to result in surface densities that hinder further hybridization.

**Table 2:** Change in Fluorescence Intensity from 2 to 24 h Hybridizations for the Three Silanes and for the 20, 25, and 30 nt Probes

Substrate	30mer (%)	25mer (%)	20mer (%)
monopodal monohydroxyl	–83	–82	–82
dipodal dihydroxyl	–31	–23	–25
dipodal monohydroxyl	–5	–23	–60

It is clear from the signal-to-background hybridization data that both dipodal silanes result in a surface functionalization that is much more stable than that of the monopodal silane. We had hypothesized that the dipodal dihydroxyl silane would outperform the dipodal monohydroxyl silane because surface functionalization with both monopodal and dipodal silanes does not result in a well-defined surface monolayer with 3 or 6 bonds to the native hydroxyl group on the glass but rather forms multilayer aggregates with polymerization between silane molecules.^58−60^ Thus, we expected that the additional hydroxyl group of the dipodal dihydroxyl silane would increase the extent of polymerization and contribute to the stabilization of the functional layer. Looking at the data for the 20mer in Table 2, we see that this is indeed the case. We hypothesize that the two primary hydroxyl groups of the dipodal dihydroxyl silane participate in cross-linking reactions during the formation of the functional layer, contributing to its stability, but at the expense of a lower density of hydroxyl groups available later for DNA synthesis, resulting in fewer surface-bound DNA probes and lower hybridization intensity. Conversely, the single, less reactive, secondary hydroxyl of the dipodal monohydroxyl silane does not contribute much to cross-linking reactions, resulting in a less-stable functionalization compared to the dipodal dihydroxyl silane. These hydroxyl groups then remain available as initiation sites for DNA synthesis, resulting in a higher initial probe density and a greater hybridization signal.

Although hybridization intensity and retention of this signal during extended exposure to warm aqueous buffers are a primary consideration in evaluating the suitability of silane functionalization, an equally important parameter is intensity homogeneity across the surface. Extracting quantitative data from high-throughput surface assays with up to circa one million probes per square centimeter requires uniform functionalization that results in signal homogeneity among equivalent surface probes synthesized at different positions across the surface. To quantify this aspect of substrate functionalization with the three silanes, we evaluated the hybridization data according to the standard deviation of the equivalent surface probes distributed across the array surface.

**Figure 3:**
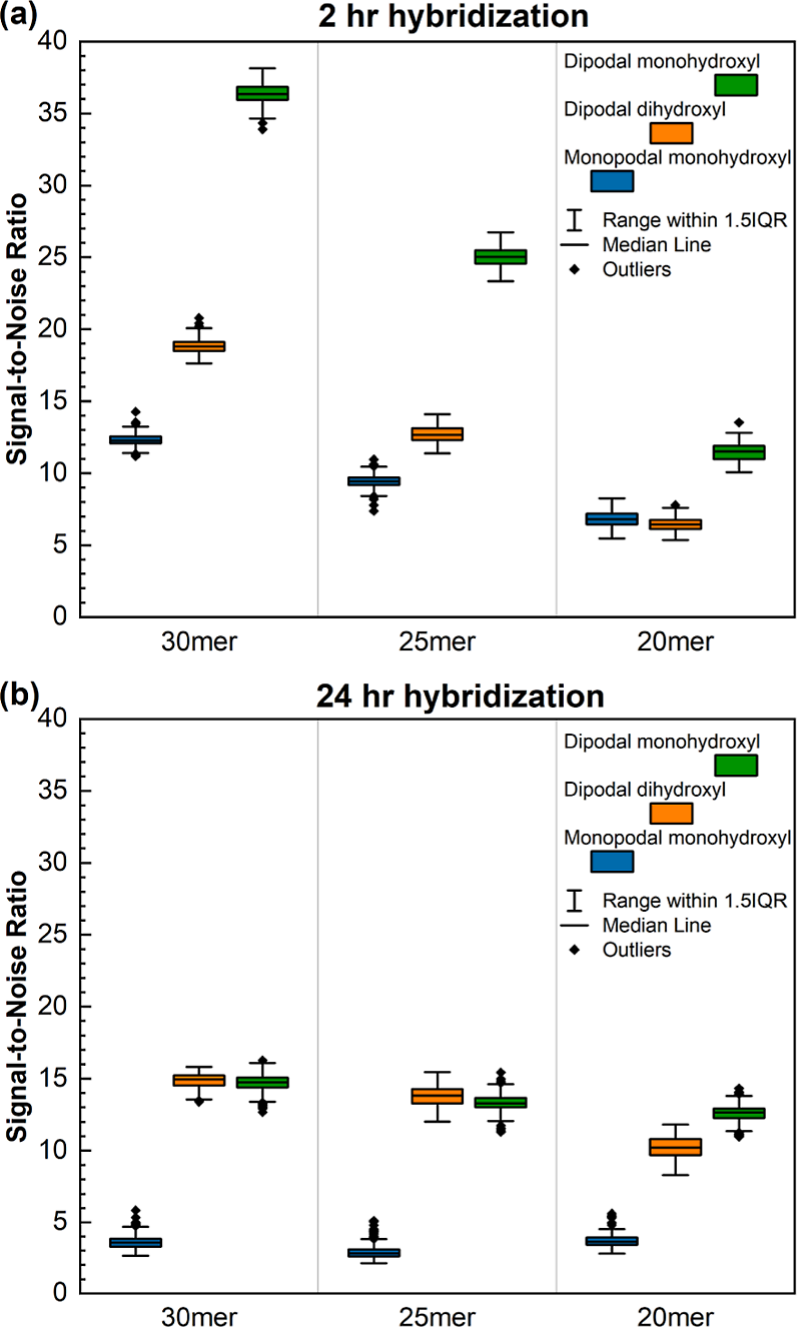
Signal-to-noise ratio of hybridization-based fluorescence intensities of 30, 25, and 20mer sequences. Intensities measured after 2 (a) and 24 h (b) of hybridization of the monopodal monohydroxyl (blue), dipodal dihydroxyl (orange), and dipodal monohydroxyl (green) silane functionalized microarrays. Noise is calculated as the standard deviation of all the replicates. Each boxplot is based on 350 replicates per sequence per array and six replicate arrays per silane.

**Figure 4:**
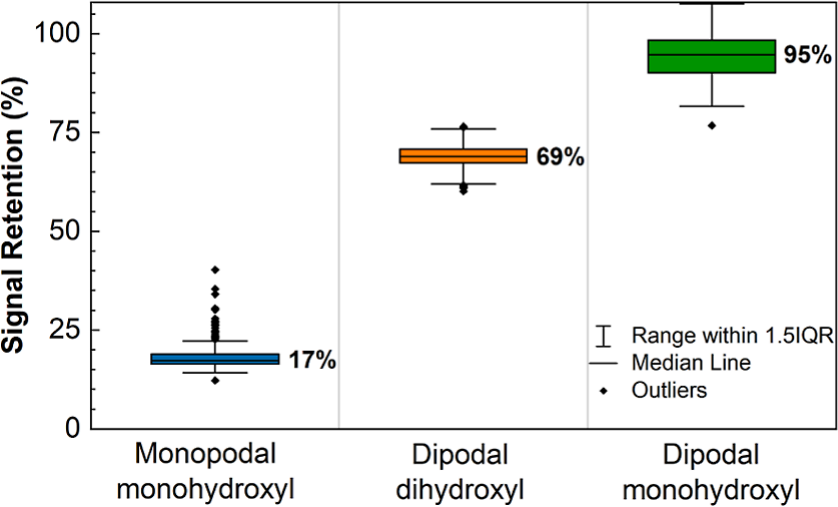
Signal retention (in percentage) calculated after 24 h hybridization. Retention is calculated in terms of signal-to-background intensity ratio of 350 replicates of the 30mer synthesized on monopodal monohydroxyl (blue), dipodal dihydroxyl (orange), and dipodal monohydroxyl (green) silane functionalized microarrays, relative to the equivalent signal after 2 h hybridization. Each boxplot is based on 350 replicates per sequence per array and six replicate arrays per silane.

These results are presented in Figure 3. In these plots, the signal-to-noise ratio is calculated as the average intensity of *n*-mers positioned at multiple locations across three replicate surfaces divided by their standard deviation. The standard deviation includes contributions from error sources independent of the surface functionalization, i.e., inhomogeneities due to the in situ photolithographic synthesis (e.g., chemical gradients and variations in light intensities reflected by the digital micromirrors), spatial patterns due to the hybridization and washing steps, and spatial artifacts introduced by the fluorescence scanner. Of these, synthesis and scanning sources contribute negligibly, but inhomogeneities due to hybridization can be significant. We took care to perform the hybridizations on the different surfaces as consistently as possible and assumed that the observed differences between the substrates could be attributed to the different surface functionalizations. The data in Figure 3 show that there are no major differences between the three substrate types in terms of homogeneity. After the 2 h hybridization, the pattern is very similar to that in Figure 2a, indicating that the differences in signal-to-noise are primarily due to differences in signal, with the two dipodal silane functionalizations clearly superior to the monopodal silane. After 24 h (Figure 3b), the two dipodal silanes perform similarly, both with signal-to-noise values approximately five times greater than the monopodal silane. Overall, it is clear that the dipodal silane surface functionalizations are superior, particularly that of the dipodal monohydroxyl. These results are summarized in Figure 4, which plots signal retention after a 24 h standard hybridization at 42 °C: 17% for the monopodal silane and 69 and 95%, for the dihydroxyl and monohydroxyl dipodal silanes, respectively. In all cases, these values are relative to the losses after 2 h, i.e., we assume no losses in the first 2 h of hybridization. This approach allows us to use standard conditions that do not result in a uniform hybridization in less than 2 h. Phillips et al.^27^ compared in situ oligonucleotide synthesis on glassy carbon and diamond thin films using the same monopodal monohydroxyl silane and found losses for the latter of approximately 95% after 24 h. We attribute their higher losses to their experimental approach, which involved rehybridizing and scanning the same array at multiple time points up to 24 h. We have observed that the additional array processing steps associated with sequential hybridizations of the same array result in greater losses. Specifically, a 24 h hybridization following a 2 h hybridization that was washed, dried, scanned, and dehybridized with warm deionized water resulted in a cumulative loss of 98% for the monopodal monohydroxyl silane and 43 and 35% cumulative losses for the dipodal monohydroxyl and dihydroxyl silanes, respectively. Otherwise, in all of our experiments, arrays were each hybridized and scanned only once, either after 2 h or after 24 h.

### 3.2 Silane-Dependent Surface Density of DNA

We hypothesized that dipodal silanes would form more bonds with the native hydroxyl groups on the glass surface. This would increase the stability of the functionalization but also result in fewer surface-bound silane molecules. The typical monopodal silanes used for surface functionalization (trialkoxysilanes), as well as their dipodal counterparts, react not only with surface hydroxyl groups but also with each other’s siloxy groups and/or functional groups to create polymeric grafted layers of varying thickness and degree of cross-linking.^58−60^ Because of the potential complexity of bond formation, the stability of the resulting polysiloxane layers may depend on both the number of bonds with the surface and the degree of cross-linking. Nevertheless, a naive model would suggest that the dipodal monohydroxyl silane would result in a more stable functionalization resulting from more bonds to the surface and a higher degree of cross-linking, but fewer functional hydroxyl groups available for DNA synthesis. This would be similar for the dipodal dihydroxyl silane, but with the doubling of functional hydroxyl groups, more would remain available for DNA synthesis. Our results (above) on the hydrolytic stability of the three functionalizations after the 2 h hybridizations indicate that the dipodal monohydroxyl silane starts out with more, rather than fewer, functional hydroxyl groups, resulting in more initiation sites for DNA synthesis and more hybridizable DNA sequences (Figure 2a), particularly in relation to the dipodal dihydroxyl silane. This is additionally surprising because the secondary alcohol of the dipodal monohydroxyl silane would be expected to be less reactive to the phosphoramidite coupling reaction in relation to the primary alcohols of the other two silanes. Because the chemical environment of Cy3 can affect its fluorescence, and because the quantum yield of fluorophores is reduced when in close proximity to each other, we used wash-off studies to quantify the hybridization density resulting from the three functionalizations, that is, the probe density of surface oligonucleotides able to bind to complementary DNA.^27^ The data from these experiments are shown in Table 3, and they indicate a larger surface density of hybridizable DNA for the dipodal silanes. Although the uncertainty in the measurement of the surface density of the dipodal dihydroxyls silanized slides is relatively high, overall, the data are consistent with the results shown in Figure 2a and indicate both that the surface fluorescence data are largely unaffected by fluorescence artifacts and that the dipodal monohydroxyl silane functionalization results in an unexpectedly high surface density of hydroxyl groups that are initiation sites for in situ DNA synthesis.

**Table 3:** Surface Density of DNA Synthesized on Three Types of Silanized Glass Slides^a^

substrate	DNA quantity (pmol/array)	DNA surface density (pmol/cm^2^)
monopodal monohydroxyl	3.1 ± 1.2	2.0 ± 0.8
dipodal dihydroxyl	6.5 ± 2.7	4.2 ± 1.7
dipodal monohydroxyl	7.6 ± 0.9	4.9 ± 0.6

a Based on Cy3-labeled complementary DNA eluted from the array after a 2 h hybridization.

### 3.3 Silane-Dependent Optimal Linker Length

**Figure 5:**
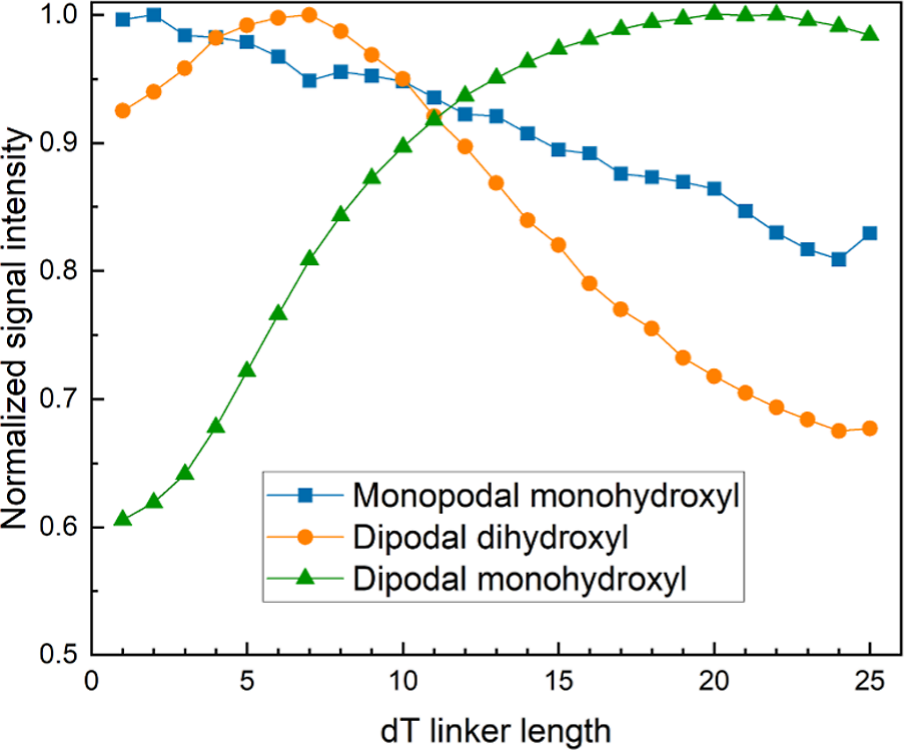
Normalized fluorescence signal from hybridization vs linker length for arrays of 25mers synthesized on glass substrates functionalized with the three silane types. Each plot is based on 350 replicates per linker length per array and two replicate arrays per silane.

Previous studies have shown that the linker (spacer) length between the surface and the functional DNA sequence (probe) influences the synthesis yield due to lower coupling efficiency near the surface, as well as the fluorescence signal in hybridization or other surface-based bioaffinity assays.^14,61,62^ The latter effect may be due to a fluorescence enhancement of commonly used cyanine dyes (e.g., Cy3) due to surface interactions.^63,64^ Because the three experimental silanes in this work are likely to result in different distances between the glass surface and their respective hydroxyl group(s), we hypothesized that each would have a distinct optimum linker length in hybridization-based surface assays. In particular, the monopodal monohydroxyl silane would require the shortest linker because its hydroxyl functional group extends about 14 Å from the silicon atom, whereas the hydroxyl group of the dipodal monohydroxyl should extend about 8 Å, assuming an intermediate siloxane binding distance on the surface (Table 1). The dipodal dihydroxyl silane would have an intermediate extension due to its hydroxyethyl functionality. Thus, we expected shorter linker requirements for monopodal monohydroxyl silane. To determine the optimal linker length, we synthesized, on glass slides functionalized with each silane, an array sharing a common 25mer probe sequence on poly dT linkers ranging in length from one to 25 nucleotides. The normalized fluorescence intensity after hybridization with the Cy3-labeled complementary sequence is shown in Figure 5. For the monopodal monohydroxyl silane, the fluorescence signal decreases slowly with length, consistent with our previous results in similar experiments.^14^ The dipodal dihydroxyl also has a more pronounced linker optimum, also at around 6 nt, followed by a fast decline. The pattern for the dipodal monohydroxyl silane is very different, with a maximum hybridization signal at a linker length of about 20 nt. While these patterns roughly match our initial expectations based on the structure of the silanes, the 20 nt linker optimal for dipodal monohydroxyl is unexpectedly long. We hypothesize that this results from the lower chemical reactivity of its secondary alcohol, resulting in the need for multiple initial coupling attempts with the dT phosphoramidite before the first coupling is successful. Since all hybridization experiments, except for the linker length determination, are based on 5 nt dT linkers, using the dipodal monohydroxyl silane with its optimal linker length would result in an even greater signal improvement relative to the other two silanes, which were tested at or near their optimal linker lengths. Another explanation for this phenomenon would be the reduced molecular crowding when extending linker length due to increased flexibility of the poly dT chain, allowing for better availability of the synthesized DNA to fluorescent complements. This would be in line with our observation on 30mer probe hybridizability (Figure 2)

### 3.4 Gene Expression Array Analysis

To test the potential of dipodal silanes for complex and particularly demanding applications in high-throughput biological assays, we synthesized high-density human gene expression microarrays on glass substrates functionalized with each of the silanes. The array of 60mer probes includes two replicates for each of three unique probes for each of ∼45,000 human transcripts plus quality control and reference sequences, for a total of 382,928 probes. These were then hybridized for 24 h with Cy3-labeled cDNA from reverse transcription of total human RNA. Since the purpose of the arrays was to evaluate the silane functionalization rather than gene expression patterns, the expression data from each array were compared with those of another array functionalized with the same silane and hybridized with another aliquot of the same cDNA. Therefore, the scatter in the data reflects experimental noise rather than over- or under-expression.

**Figure 6:**
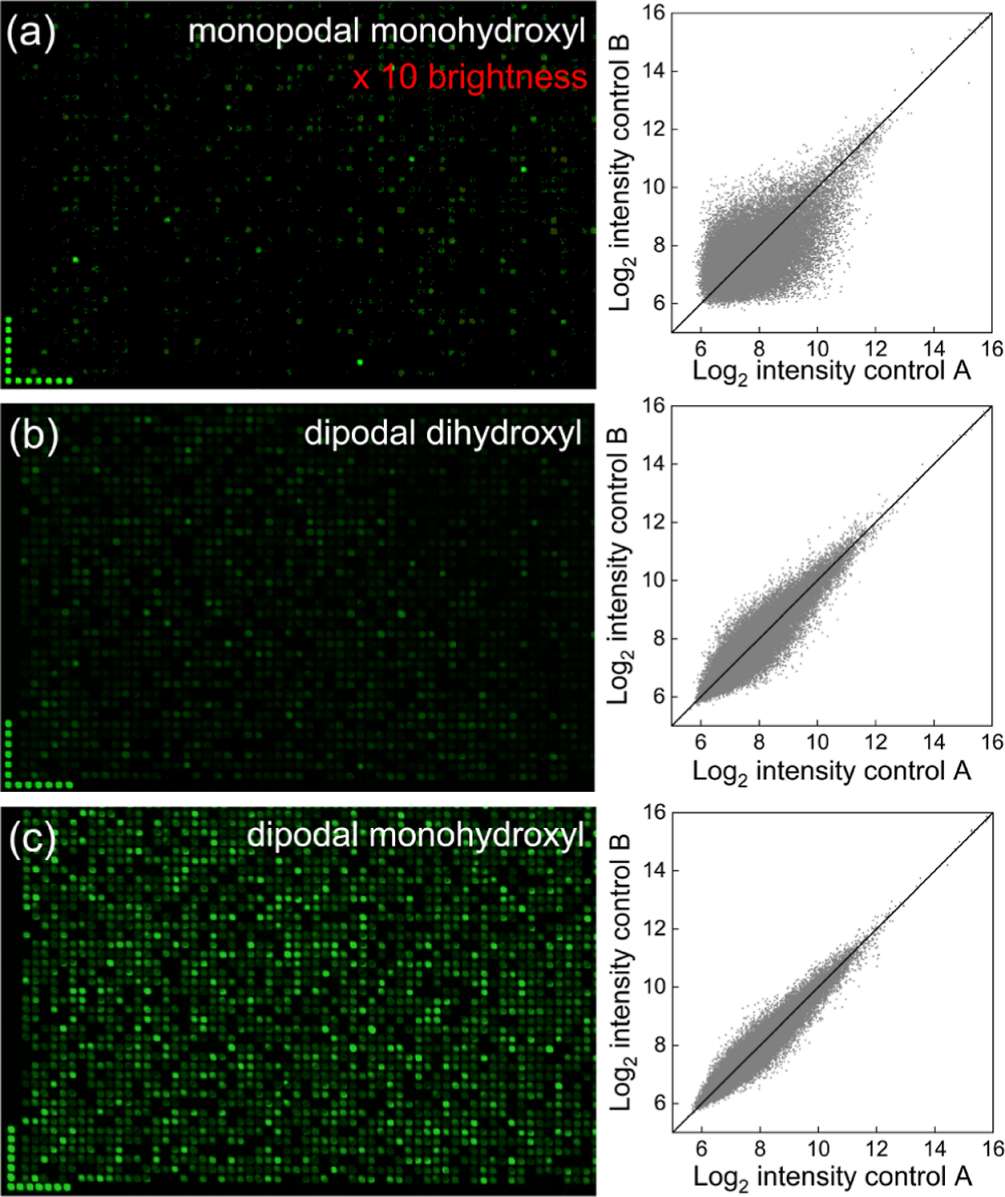
Gene expression arrays synthesized on glass substrates functionalized with (a) the monopodal monohydroxyl silane, (b) the dipodal dihydroxyl silane, and (c) the dipodal monohydroxyl silane. The left column shows a detail (∼0.5% of each array) of representative fluorescence scanned images. Each spot measures 10 × 10 μm. The right column shows the respective scatterplots of RMA-processed data from the arrays. Each scatterplot is based on two arrays (control A and control B) per silane; each expression value is the average of three unique 60mer probes per gene.

Figure 6 shows representative images from the hybridized gene expression arrays synthesized on substrates functionalized with each of the three silanes as well as the corresponding log_2_ scatterplots. The three image details closely match the intensity profiles shown in Figure 1b for fluorescence intensities after 24 h hybridizations of low complexity arrays of 20 through 30mers. In particular, the surface functionalization with the dipodal monohydroxyl silane results in a significantly brighter image in comparison with that of the dipodal dihydroxyl silane, which in turn is about ten times brighter than that from the monopodal monohydroxyl silane array. The corresponding scatter plots reflect this pattern, demonstrating that the loss of probes due to silane hydrolysis, particularly for the monopodal silane, results in a large loss of signal and a concomitant decrease in the signal-to-noise ratio of the assay. To quantify the improvement in the signal-to-noise ratio of the gene expression data resulting from the use of dipodal silanes, we calculated the root-mean-square error (RMSE) of each of the 51,957 points on the scatter plots in Figure 6 relative to their error-free value, represented by the diagonal line. These RMSE values are shown in Table 4, along with the mean intensities (nonnormalized) of the corresponding genomic and synthetic spike-in controls. Although the two metrics cannot be directly compared, the RMSE values are consistent with the signal-to-noise ratios for the 30mers plotted in Figure 3b, also for 24 h hybridizations, with both dipodal silanes being significantly better able to discriminate signal from noise relative to the monopodal silane. We also note that for direct comparison purposes, the hybridization data shown in Figures 1–4 and 6 are all based on arrays synthesized with dT_5_ linkers. An additional signal enhancement of about 30% in the case of substrates functionalized with the dipodal monohydroxyl silane can additionally be achieved by increasing the linker length to 20 nt, as shown in Figure 5, albeit at the expense of a few minutes of additional synthesis time.

**Table 4:** Root Mean Square Error and Mean Fluorescence Intensity for Genomic and Quality Control Spike-In Probes for the Gene Expression Arrays Synthesized with the Three Silanes

	monopodal monohydroxyl	dipodal dihydroxyl	dipodal monohydroxyl
root mean square error (RMSE)	0.88	0.42	0.30
RMSE relative to monopodal silane	1	0.48	0.34
mean genomic probe intensity	31	123	337
mean spike-in probe intensity	1036	7998	20,476

## 4 Conclusions

In this work, we have shown that dipodal silanes greatly increase the hydrolytic stability of glass surface functionalizations and that these functionalizations are compatible with the in situ DNA synthesis of complex DNA arrays. Whereas the monopodal silane results in the loss of about 83% of the DNA in the course of a 24 h hybridization, the dipodal dihydroxyl silane substrate loses 31% and the dipodal monohydroxyl only 5%. The stability of the dipodal silanes on glass is therefore comparable to hydroxyl-functionalized glassy carbon, but less stable than hydroxyl-functionalized nanocrystalline diamond thin films while retaining the advantages of glass and the accessibility and versatility of silane-based functionalization. The preservation of DNA on dipodal silanes leads to about a 2- to 4-fold increase in the signal-to-noise ratio—in 24 h hybridization experiments—relative to the monopodal silane. Although both dipodal silanes performed much better than the monopodal silane, the dipodal monohydroxyl silane appears to be a marginally better choice than the dipodal dihydroxyl silane for hybridizations up to 24 h and at moderate temperatures (42 °C), resulting in a higher signal and in a similar or better signal-to-noise ratio in all experiments. Nevertheless, the dipodal dihydroxyl silane appears to be more hydrolytically stable and may perform better for longer incubation times and/or higher temperatures.
